# Capturing the antibiotic resistome of preterm infants reveals new benefits of probiotic supplementation

**DOI:** 10.1186/s40168-022-01327-7

**Published:** 2022-08-26

**Authors:** Allison K. Guitor, Efrah I. Yousuf, Amogelang R. Raphenya, Eileen K. Hutton, Katherine M. Morrison, Andrew G. McArthur, Gerard D. Wright, Jennifer C. Stearns

**Affiliations:** 1 Department of Biochemistry and Biomedical Sciences, McMaster University, Hamilton, Canada; 2 Michael G. DeGroote Institute for Infectious Disease Research, McMaster University, Hamilton, Canada; 3 David Braley Centre for Antibiotic Discovery, McMaster University, Hamilton, Canada; 4 Department of Pediatrics, McMaster University, Hamilton, Canada; 5 Department of Obstetrics & Gynecology, McMaster University, Hamilton, Canada; 6 The Baby & Mi and the Baby & Pre-Mi Cohort Studies, Hamilton, Canada; 7 Department of Medicine, McMaster University, Hamilton, Canada; 8 Farncombe Family Digestive Health Research Institute, McMaster University, Hamilton, Canada

**Keywords:** Probiotics, Preterm infants, Gut microbiota, Antibiotics, Resistome, Targeted capture

## Abstract

**Background:**

Probiotic use in preterm infants can mitigate the impact of antibiotic exposure and reduce rates of certain illnesses; however, the benefit on the gut resistome, the collection of antibiotic resistance genes, requires further investigation. We hypothesized that probiotic supplementation of early preterm infants (born < 32-week gestation) while in hospital reduces the prevalence of antibiotic resistance genes associated with pathogenic bacteria in the gut. We used a targeted capture approach to compare the resistome from stool samples collected at the term corrected age of 40 weeks for two groups of preterm infants (those that routinely received a multi-strain probiotic during hospitalization and those that did not) with samples from full-term infants at 10 days of age to identify if preterm birth or probiotic supplementation impacted the resistome. We also compared the two groups of preterm infants up to 5 months of age to identify persistent antibiotic resistance genes.

**Results:**

At the term corrected age, or 10 days of age for the full-term infants, we found over 80 antibiotic resistance genes in the preterm infants that did not receive probiotics that were not identified in either the full-term or probiotic-supplemented preterm infants. More genes associated with antibiotic inactivation mechanisms were identified in preterm infants unexposed to probiotics at this collection time-point compared to the other infants. We further linked these genes to mobile genetic elements and *Enterobacteriaceae*, which were also abundant in their gut microbiomes. Various genes associated with aminoglycoside and beta-lactam resistance, commonly found in pathogenic bacteria, were retained for up to 5 months in the preterm infants that did not receive probiotics.

**Conclusions:**

This pilot survey of preterm infants shows that probiotics administered after preterm birth during hospitalization reduced the diversity and prevented persistence of antibiotic resistance genes in the gut microbiome. The benefits of probiotic use on the microbiome and the resistome should be further explored in larger groups of infants. Due to its high sensitivity and lower sequencing cost, our targeted capture approach can facilitate these surveys to further address the implications of resistance genes persisting into infancy without the need for large-scale metagenomic sequencing.

Video Abstract

**Supplementary Information:**

The online version contains supplementary material available at 10.1186/s40168-022-01327-7.

## Background

Preterm infants (born < 37-week gestation) have an immature gut microbiome that is shaped by various factors, including the immaturity of the gastrointestinal tract at birth, maternal and postnatal antibiotic exposure, delivery mode, and feeding method [1–8]. Preterm infants often have reduced gut microbial diversity compared to full-term infants. Their microbiota can be dominated by a few potentially pathogenic bacteria, including *Enterococcus faecalis*, *Staphylococcus epidermidis*, *Klebsiella pneumoniae*, *Escherichia coli*, and *Pseudomonas aeruginosa* [3, 6, 9, 10]. The initial colonizers of the preterm infant gut have been linked to the environment, including the neonatal intensive care unit (NICU) [11, 12]. The potential exposure to multidrug-resistant (MDR) strains, their persistence in the infant gut, and possible transfer of antibiotic resistance genes (ARGs) in this hospital niche are of concern [13, 14].

Due to their overall immune immaturity, preterm infants are at high risk of infection and can be exposed to antibiotics for prolonged periods, usually as empiric treatment for suspected sepsis [15]. Among the most frequently used medications in the NICU are the antibiotics ampicillin, gentamicin, amikacin, and vancomycin [15, 16]. The consequences of antibiotic exposure on preterm infants’ gut microbiota and resistome, or ARG content, have been explored [8, 17]. Exposure to various broad-spectrum antibiotics not only reduces the overall diversity of bacteria in the infant gut but also can select for pathogenic *Enterobacteriaceae* and reduce beneficial organisms, including *Bifidobacteriaceae* [18–21]. This change in microbial diversity is also related to a selection for MDR strains and promotes persistence of ARG-carrying bacteria over time [13, 18, 21–23].

The intestinal immaturity of preterm infants, the reduced microbial diversity in the gut, and frequent exposures to antibiotics increase the risk of necrotizing enterocolitis (NEC) for these infants [24–26]. NEC is a disease that affects around 7% of very low birth-weight infants, is associated with longer hospital stays, and has a high mortality rate (15–30%) [24, 27]. Probiotics have been shown to reduce the incidence of NEC, sepsis, and mortality in preterm infants, in addition to reducing the impact of extensive antibiotic exposure on the gut microbiota [2, 24, 28–37].

A higher prevalence of *Bifidobacterium* species in the infant gut is associated with reduced colonization by taxa commonly associated with antimicrobial resistance (AMR) such as *Enterobacteriaceae* [1, 38–40]. By association, we hypothesized that a probiotic supplement containing *Bifidobacterium* species will reduce the diversity of ARGs detected in the preterm infant gut. A few studies have focused on probiotics and the resistome of preterm infants and reported that *Bifidobacterium*-containing probiotics reduced the abundance of MDR bacteria and their associated ARGs [41–43]. These studies used either polymerase chain reaction (PCR)-based approaches [42] or shotgun metagenome sequencing [41, 43] to characterize the resistome. Because ARGs usually represent less than 1% of a metagenome, the latter approach of shotgun sequencing requires a high number of sequencing reads, which becomes expensive for longitudinal cohort studies [44]. PCR-based resistome analyses typically only target a few antibiotic resistance genes of interest [45]. Our previous work showed how a targeted capture probe set of over 37,000 nucleotide baits designed against 2000 antibiotic resistance genes is superior to shotgun sequencing for surveying the resistome [46]. We employed this probe set to profile ARGs of preterm infants supplemented with probiotics and highlight the reduction of these genes commonly associated with pathogenic bacteria.

A subset of preterm infants from the Baby and Preterm Microbiota of the Intestine Cohort Study (Baby & Pre-Mi) at McMaster University received a commercial probiotic supplement (FloraBABY, Renew Life Canada) that contained four species of *Bifidobacterium* and one *Lactobacillus* species [47]*.* We have previously shown that at term age, the gut microbiota of these preterm infants supplemented with this probiotic was more similar to that of 10-day-old healthy infants born full term (> 37-week gestation) [4, 5, 47]. In this study, we assessed the resistome of the gut microbiota in a subset of samples from preterm and full-term infants from the Baby & Pre-Mi and Baby & Mi studies [4, 5, 47], including stool samples collected during hospitalization and at follow-up visits up to 5-month corrected age from 8 preterm infants who were supplemented with the probiotic in hospital (PS), 13 preterm infants that were not supplemented with the probiotic during hospitalization (NS) [47], and stool samples from nine 10-day-old full-term (FT) infants that did not receive probiotics or antibiotics [4, 5]. DNA extracts were prepared for Illumina sequencing and enriched for ARGs using targeted capture [46]. After enrichment and sequencing, the Resistance Gene Identifier’s (RGI) metagenomic feature was used to map reads to the Comprehensive Antibiotic Resistance Database (CARD) [48]. ARGs were compared across patient cohorts and study time-points to detect differences in the resistome after probiotic supplementation. We highlight differences in the diversity of ARGs rather than individual ARG abundances, given that even rare ARGs in the microbiome can provide selection for antibiotic-resistant bacteria and the future implications of ARG persistence on preterm infant health are unknown.

## Methods

### Study participants and sampling

A detailed description of the study participants and design can be found in Yousuf et al. [47] and Stearns et al. [5]. Four stool samples were chosen for preterm infants in our study, based on time-point sample availability and distribution to provide a longitudinal survey spanning their time spent in hospital up to 5 months of age. The last sample collected in hospital before the infant was discharged or they reached their expected due date (i.e., term age) is referred to as the in-hospital time-point. The other samples included are the first study visit (visit 1) that took place as close to term age as possible and subsequent samples collected at around 6-week (visit 2), 12-week (visit 3), and 5-month (visit 4) corrected age (with corrected age referring to age of the infant from the expected due date). Postmenstrual age (PMA) in weeks at the time of sample collection was calculated as the sum of gestational age at birth (based on the expected due date and infant birth date) and postnatal age.

Part way through the Baby & Pre-Mi study (November 2017), the McMaster Children’s Hospital NICU changed their policy such that the probiotic FloraBABY (Renew Life Canada, Brampton, ON, Canada) was routinely given to infants born at less than 34-week gestation or weighing less than 2 kg. This probiotic contains 0.5 g (2 billion CFU bacteria) per single-dose sachet, including the following: *Bifidobacterium breve* (HA-129), *Lactobacillus rhamnosus* (HA-111), *Bifidobacterium bifidum* (HA-132), *Bifidobacterium longum* subsp*. infantis* (HA-116), and *Bifidobacterium longum* subsp. *longum* (HA-135). For our resistome analysis, infants born early preterm (< 32-week gestation) that were admitted to the NICU and had samples available in hospital and at around term age were studied. This includes 8 probiotic-supplemented (PS) preterm infants born at an average gestational age of 28.14 weeks and 13 not supplemented (NS) preterm infants born at an average of 27.49 weeks. The PS infants were exposed to the probiotic FloraBABY for an average of 8.27 weeks (Table 1). One PS infant (PS4) continued supplementation with the probiotic BioGaia, which contains *Limosilactobacillus reuteri* strain DSM 17938, throughout visit 1 and visit 2 after stopping FloraBABY administration post-discharge from hospital. Two NS infants (NS3 and NS4) received the probiotic BioGaia between visit 3 and visit 4 for an unknown duration. As a comparator to the term age (visit 1) sample collected from preterm infants, a 10-day stool samples from 9 full-term (FT) infants from the Baby & Mi study were included (Table S1, Additional file 1) [5]. These full-term infants had not received probiotics or antibiotics prior to stool collection. From our chosen set of preterm infants (*n* = 21), a subset of samples from PS (*n* = 6) and NS (*n* = 6) infants were matched for antibiotic exposure and sample availability (Table S2, Figs. S2, S3, Additional file 1). We present the results from this subset alongside the results from the entire cohort to determine if the results were replicated.

**Table 1:** Characteristics of infant cohorts and samples used in this study

	NS preterm (*n* = 13)	PS preterm (*n* = 8)	*p*-value
Gestational age at birth, weeks	27.49 ± 2.03	28.14 ± 1.54	0.47
Probiotic exposure, weeks	0.00	8.27 ± 3.19	**< 0.0001**
Antibiotic exposure during sample collection (types and number of infants exposed)	Amo(1), Amp(13), Az(2), Cefa(3), Cefo(5), Cefu(1), Cl(5), G(13), Mer(2), Met(1), T(1), V(5)	Amp(6), Cefa(1), Cefo(2), Cl(3), G(6), Met(2), V(1)	N/A
Antibiotic exposure, weeks	1.98 ± 1.83	1.11 ± 1.20	0.20
Inhospital sample, weeks in PMA (*N*)	37.20 ± 3.80 (12)	37.86 ± 1.69 (3)	0.66
Visit 1 sample, weeks in PMA (*N*)	41.96 ± 2.25 (10)	42.63 ± 1.69 (8)	0.52
Visit 2 sample, weeks in PMA (*N*)	46.83 ± 1.75 (9)	46.43 ± 0.50 (5)	0.90
Visit 3 sample, weeks in PMA (*N*)	52.29 ± 2.45 (7)	54.21 ± 1.85 (6)	0.18
Visit 4 sample, weeks in PMA (*N*)	62.46 ± 2.56 (10)	59.68 ± 0.62 (4)	0.07

PMA is the postmenstrual age in weeks, and SD is the standard deviation. The data are presented as mean ± SD. *P*-values < 0.05 using Student’s *t*-test or Mann-Whitney were considered to be statistically significant

### 16S rRNA gene profile analysis

The amplicon sequence variant (ASV) table from [47, 49] representing bacterial 16S rRNA gene v3 region amplicons was analyzed by plotting the relative abundance of ASVs for each full-term infant at 10 days and the subset of preterm infants included in our study at all available collection time-points using R. ASVs were grouped together as one category (< 1% abundance) if they represented less than 1% of the total relative abundance across all infants.

### DNA library preparation, enrichment, and sequencing

DNA extracted from stool for the previous study [5, 47, 50] was used here. Library preparation and enrichment for ARGs were performed as described in Guitor et al. [46]. When available, up to 500 ng of dsDNA was used for library preparation with the NEBNext Ultra II dsDNA library kits (Additional files 2, 3). After library preparation, a High Sensitivity DNA ScreenTape Analysis (Agilent Technologies) was performed to estimate the DNA available for enrichment. For most samples, at least 100 ng of DNA was available for enrichment (Additional file 2). All samples were enriched for 24 h at 65 °C using a probe set of 37,826 probes designed to target over 2000 ARGs. After 3 rounds of washing of the streptavidin beads, 12.5 μL of captured DNA was amplified by PCR for 14 cycles, purified using KAPA Pure Beads, and then eluted in 30 μL of 10 mM Tris HCl, pH 8.3. Enriched libraries were quantified by quantitative PCR (qPCR) and then pooled in equimolar ratios. Sequencing was performed by the Farncombe Metagenomics sequencing facility at the McMaster University on an Illumina HiSeq or MiSeq with 2 × 250 bp sequencing chemistry with a targeted depth of 250,000 clusters per library.

### Analysis of targeted capture sequencing data

Paired sequencing reads were trimmed using *skewer* version 0.2.2 [51, 52] and deduplicated using *dedupe.sh* from BBMap version 38.57 [53]. Reads were then subsampled to 50,000 pairs, or 100,000 paired reads total, using the *sample* command from seqtk version 1.3 [54]. Using the beta read mapping to CARD (RGI *bwt*) feature of RGI version 5.1.1 [55], reads were mapped to a combined reference of 179,050 nucleotide sequences from CARD (nucleotide sequences, protein homolog model, version 3.1.0) and the Resistomes & Variants database version 3.0.7 using bowtie2 version 2.3.5.1 [56, 57]. Both databases are available for download [58]. We additionally used RGI’s beta feature for the k-mer prediction of pathogen of origin for AMR genes or reads (RGI *kmer_*query) with the default 61-mer database, to predict bacterial species that may harbor an ARG. Lastly, using in-house scripts, we generated de novo assemblies of the enriched metagenomes using the *metaSPAdes* option in SPAdes v. 3.13.1 [59, 60]. Resistance genes were predicted from these assemblies using the *main* feature of RGI version 5.1.1 and CARD (version 3.1.0–2702 nucleotide sequences). Detailed code is available at https://github.com/AllisonGuitor/AMR-metatools. An important distinction to note is that RGI’s *bwt* read-mapping algorithm in version 5.1.1 is unable to detect resistance conferred by point mutation in chromosomally encoded ARGs, whereas RGI *main* can predict resistance via this mechanism.

The RGI *bwt* results for each infant were filtered for genes with at least 100 mapped reads. If a de novo assembly was successfully generated, all perfect and strict predicted genes from the RGI analysis were included in downstream analyses. These results were then combined across all infants. Significant differences between infant groups were determined by unpaired *t*-test in GraphPad Prism version 9.0.1, with a *p*-value cutoff of below 0.05. To assess whether the types of resistance genes differ between infant groups, we further categorized the genes identified through RGI *bwt* based on their AMR resistance mechanisms and gene families in CARD. Unique genes identified in each infant cohort in the RGI *bwt* analysis and the de novo assembly + RGI *main* analysis were compared. Venn diagrams were generated using BioVenn [61]. To compare ARGs that we differentially detected in one group of infants compared to the others, we excluded AMR gene families that were present in all cohorts at the visit 1 time-point (term age for preterm and 10 days of age for full-term infants). For the longitudinal analysis, we removed AMR gene families that were present in 4/5 time-points in both preterm infant cohorts, as these likely represent the general resistome of preterm infants.

### Mobile genetic element detection and bacterial host identification

Given the nature of our probe set design and method, the genomic context surrounding targeted resistance genes can also be captured. This analysis relies on assembling contigs from the targeted capture data and successfully predicting open reading frames (ORFs). Therefore, in some cases, a particular gene might not be detected. In many cases, large enough contigs were obtained to predict an ARG through RGI *main* and to annotate neighboring genes using Prokka version 1.14.5 [62]. Only contigs greater than 1.2 kb with perfect or strict hits from CARD’s protein homolog model were considered. Contigs greater than 1.2 kb containing ARGs were analyzed using *mob_recon* from MOB-suite v3.0.1 [63, 64] to predict potential plasmid sequences and mobile genetic elements (MGEs). In addition to these potentially mobile ARGs, we selected contigs with AMR gene families that were unique to one infant cohort when comparing preterm infants at term to FT infants at 10 days of age or to one infant cohort when comparing preterm infants up to 5 months of age. This included the following: *aac(3)*, *ant(2″)*, *ant(4′)*, *aph(3′)*, *arr-3*, *bla*_ACT_, *bla*_CTX-M_, *bla*_CblA_, *bla*_DHA_, *bla*_LEN_, *bla*_MIR_, *bla*_MOX_, *bla*_OXA_ *kdpDE*, *streptothricin acetyltransferase*, *tetracycline inactivation enzymes*, *vancomycin resistance genes*, and *streptogramin vat acetyltransferase*. We retrieved the most similar nucleotide hit of these contigs using Nucleotide BLAST (blastN) [65] with the nonredundant nucleotide collection in NCBI [66]. We also recorded the pathogen-of-origin prediction by RGI *kmer_query*, and results from read mapping to CARD’s Resistomes & Variants database which provides potential hosts for each ARG based on reported sequencing data in NCBI [67]. From the blastN, RGI *kmer_query*, CARD’s Resistomes & Variants database, and the *mob_recon* analysis, a consensus prediction of a bacterial host for each ARG was inferred if at least 2 of the results agreed. Annotated contigs containing unique ARGs of interest were compared using clinker version 0.0.21 [68].

### Negative controls sequencing

We included 12 negative controls throughout our study to account for potential contamination from reagents and laboratory environment. The libraries were analyzed via a High Sensitivity DNA ScreenTape Analysis (Agilent Technologies) and quantified by qPCR as described previously [46]. Only 3 of the negative controls displayed signatures of DNA sequencing libraries, and one had sufficient concentration for sequencing (Additional file 5). This sample was sequenced on an Illumina MiSeq run (2 × 250 bp chemistry) separately from other libraries generated in this study. The unique index combination corresponding to the negative control library did not generate any read data.

## Results

### Exposure of preterm infants to various antibiotics and probiotics in early life

**Fig. 1:**
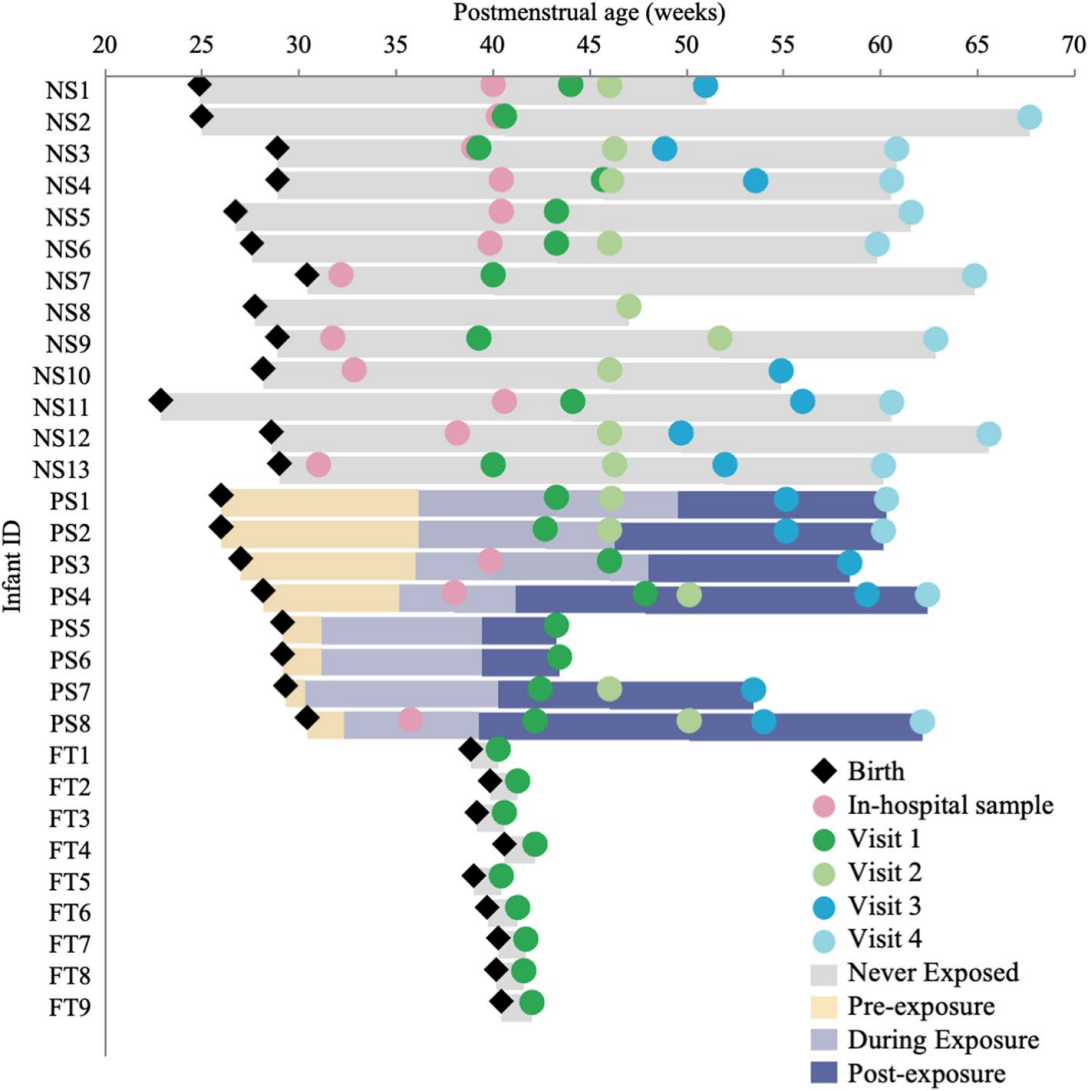
Sample collection and probiotic exposure of preterm infants. Timelines from birth to final sample collection for all infants are included in this study. The duration of exposure to probiotics (lavender bar) and timing of sample collection in relation to postmenstrual age in weeks are shown for non-probiotic-supplemented (NS), probiotic-supplemented (PS) preterm infants, and full-term (FT) infants

All preterm infants included in this study were treated with ampicillin and gentamicin, except two PS infants that did not receive antibiotics during sample collection. Many received up to five different antibiotics within the first 10 weeks of life (Fig. S1, Additional file 1). On average, NS infants received more prolonged doses of antibiotics (1.98 weeks compared to 1.11 weeks for PS infants; however, the difference was not profound), with one NS infant receiving a total of 5.86 weeks of antibiotic dosing between birth and 5-month corrected age (Table 1). No PS infant received antibiotics after beginning probiotic administration during the study period. A timeline of sample collection and probiotic administration is shown in Fig. 1. Three of the preterm infants were still receiving probiotics during the visit 1 or term age time-point, and PS4 continued to receive BioGaia at the visit 1 and visit 2 time-points. In previous work, the impact of probiotics on the gut microbiota of this cohort of infants is described in detail [47]. Our study found that bacteria belonging to the family Bifidobacteriaceae are present at higher relative abundances at earlier collection time-points in PS infants than in NS infants, and that *Enterobacteriaceae* and *Clostridiaceae* dominate the gut microbiota of NS infants (Fig. S4, Additional file 1).

### Similar number of antibiotic resistance genes in preterm and full-term infants at an early age

We first wanted to compare the resistome of preterm infants to full-term infants at an early age. We found that neither preterm birth nor probiotic exposure in preterm infants resulted in significant differences in the number of ARGs recovered from these infant gut microbiomes at the term age as compared to full-term infants at 10 days of age (Fig. S5 B–C, Additional file 1). Given the variability in the number of samples between the NS, PS, and FT infants, and differences in antibiotic treatments of the preterm infants, we reanalyzed our results in a subset of preterm infant samples matched for time-point and antibiotic administration along with the FT infants. Again, we found no significant differences in the number of ARGs between the infant groups (Fig. S6 B–C, Additional file 1). To ensure our results were not biased, we compared the percentages of sequencing reads that map to ARGs in CARD between infant groups. We found a significantly higher number of reads mapping in the FT compared with the NS infants at visit 1 (10 days/term age) (*P* = 0.0014; *P* = 0.0318, Figs. S5A and S6A, Additional file 1). The higher percentage of mapped reads to CARD did not correspond to increased numbers of ARGs in the full-term infants. Therefore, percentage of mapped reads to CARD cannot be used as a measure of ARG load after enrichment as it is in shotgun sequencing but is instead used as a measure to determine whether enrichment was successful [41].

### Preterm infants not supplemented with probiotics have a greater diversity of antibiotic resistance genes

**Fig. 2:**
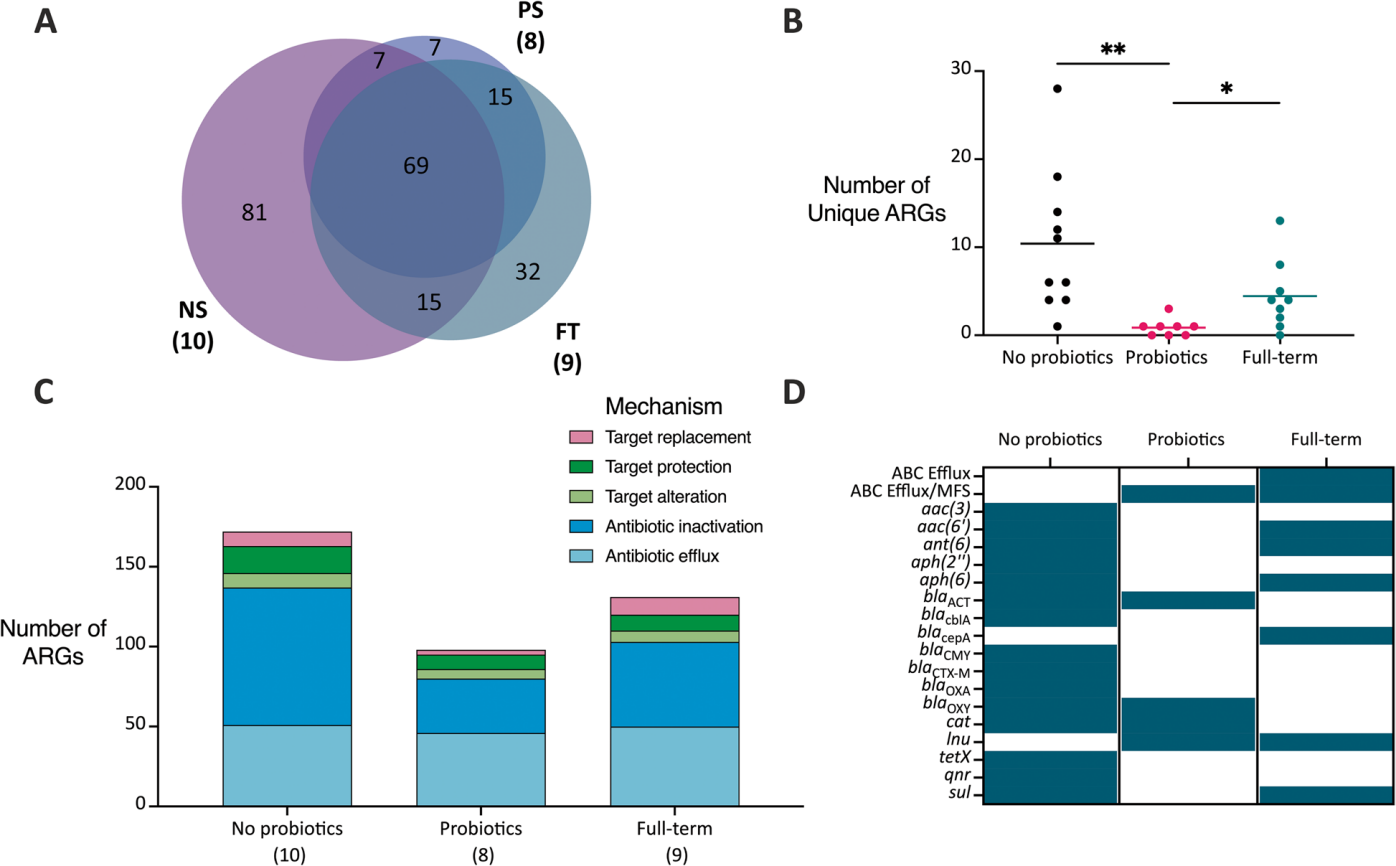
Differences in the resistome identified through RGI *bwt* in infants at visit 1. Reads were mapped to CARD using bowtie2, and antibiotic resistance genes with at least 100 reads were reported. The data presented is from the full set of preterm and full-term infants and at visit 1. A) Unique and overlapping ARGs identified in each infant group. The number of infant samples included in each is shown next to the sample type. B) The number of unique ARGs identified in each infant. Significant differences are denoted by a line and asterisk(s) above the groups that were compared (*P* = 0.0047 for NS vs PS, *P* = 0.0262 for PS vs FT). C) A breakdown of the mechanisms of antibiotic resistance identified in each infant group. The number of infant samples included in each is shown next to the sample type. D) The presence or absence of selected AMR gene families in each infant group. A teal box indicates that at least one gene from that AMR gene family was identified in any of the infant samples (*NS* = not supplemented preterm, *PS* = probiotic-supplemented preterm, and FT = full-term infants)

We next compared the types of ARGs found in each infant group at an early age (visit 1). In both the read-mapping RGI *bwt* and de novo assembly with RGI *main* analysis approaches, over 200 ARGs were identified (226 for RGI *bwt* and 243 for RGI *main*) in all infants (Figs. 2A, S7A, Additional file 1). Many of these ARGs (81 for RGI *bwt* and 94 for RGI *main*) were only ever identified in the NS infants (Figs. 2A, S7A, Additional file 1). We then looked at the number of ARGs at the individual level that was unique to their given infant group and found significantly fewer unique ARGs in each PS infant compared to the NS and FT infants (*P* = 0.0047 for NS vs PS, *P* = 0.0262 for PS vs FT; Figs. 2B, S7B, Additional file 1). Many of the NS infants had more than 10 ARGs that were not identified in the two other infant groups (Figs. 2B, S7B, Additional file 1). We found that probiotic exposure in preterm infants resulted in a reduced number of unique ARGs in the infant gut microbiome as compared to other preterm infants at the term age and full-term infants at 10 days of age.

When classified under their respective AMR mechanism, we identified more antibiotic inactivation genes in the NS infant group compared to the other infants (Figs. 2C, S7C, Additional file 1). Finally, we refined our results to the AMR gene family level and determined the presence of AMR gene families in the infant groups (Figs. S8A, S9A, Additional file 1). Various AMR gene families were found uniquely in the NS infants including aminoglycoside-modifying enzymes (*aac(3)*, *aph(2″)*) and beta-lactamases (*bla*_CTX-M_, *bla*_CMY_, *bla*_OXA_) (Figs. 2D, S7D, Additional file 1). Again, when looking at the subset of preterm infant samples matched for time-point and antibiotic exposure along with the full-term infants, we found similar results to those above (Figs. S8B, S9B, S10, S11, Additional file 1). We found that probiotic exposure in preterm infants resulted in a reduced diversity of ARGs in the infant gut microbiome as compared to other preterm infants at the term age and full-term infants at 10 days of age.

### Probiotics reduce the diversity of the preterm gut resistome up to 5 months of age

**Fig. 3:**
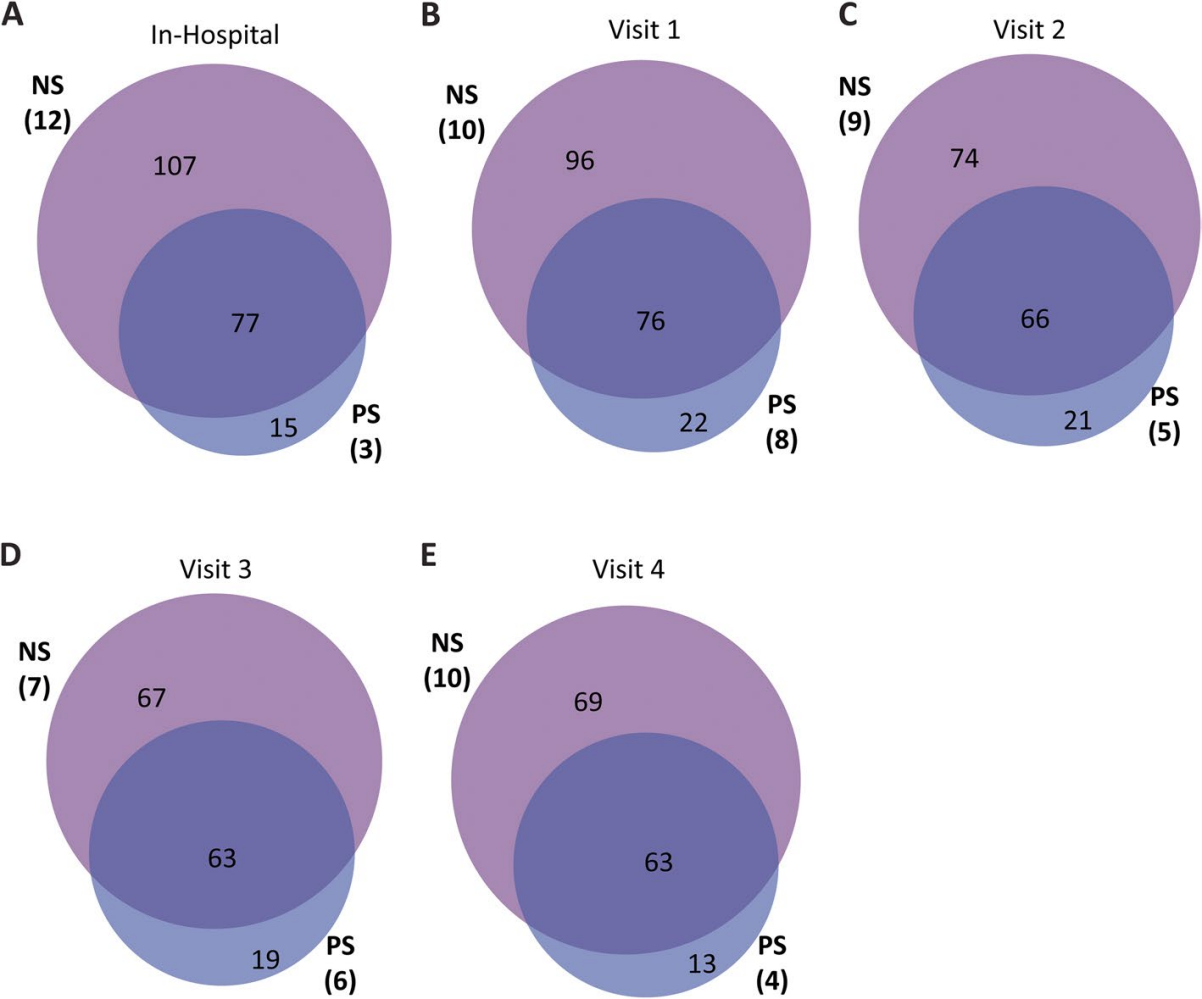
Number of unique genes in preterm infants at various time-points. These gene counts are from mapping reads to CARD using bowtie2 and counting the number of genes with at least 100 reads. Data are from NS and PS infants at the inhospital collection (A), visit 1 (B), visit 2 (C), visit 3 (D), and visit 4 (E) time-points. The number of infants included in each time-point is indicated (NS = non-probiotic-supplemented preterm, PS = probiotic-supplemented preterm)

**Fig. 4:**
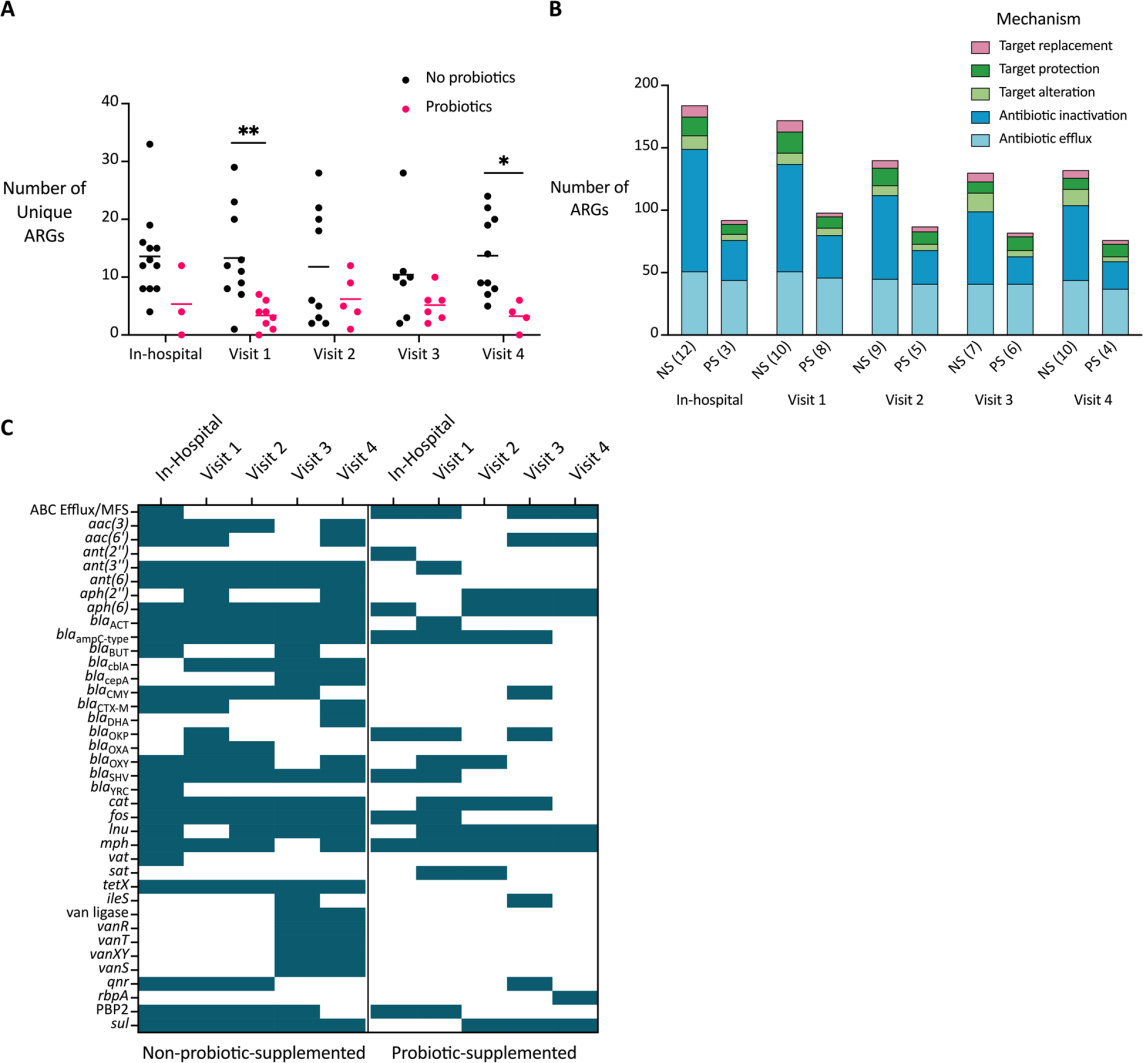
Unique ARGs, mechanisms, and families in preterm infants up to 5 months of age. Reads were mapped to CARD using bowtie2, and ARGs with at least 100 reads were reported. The data presented is for all preterm infants at all visits. A) The number of unique ARGs identified in each infant. Significant differences are denoted by a line and asterisk(s) above the groups that were compared (*P* = 0.0052 for visit 1, *P* = 0.0144 for visit 4). B) The number of ARGs identified in each infant group classified by resistance gene mechanism. The number of infant samples included in each is shown next to the sample type (NS = non-probiotic-supplemented preterm, PS = probiotic-supplemented preterm). C) A selected subset of detected AMR gene families in preterm infants. A teal box indicates that at least one gene from that AMR gene family was identified in any of the infants at that time-point

Next, we sought to compare the impact of probiotics on the resistome of preterm infants up to 5 months of age. Apart from the differences at visit 1 noted above, we found no significant differences in the percentage of reads mapping on target or the number of ARGs detected at any time-point between the NS and PS infants (Figs. S5DEF, S6DEF, Additional file 1). We did, however, identify more ARGs in the NS infants as a group compared to the PS infants at all study time-points up to 5 months of age (Figs. 3, S12, Additional file 1). These results were recapitulated in the matched subset of infants except at the 12-week corrected age (visit 3) where ~50% of the unique genes (21/44) were identified in one PS infant (results not included) highlighting the potential for large individual variation in the resistome among infants (Figs. S13, S14, Additional file 1). Again, we looked at the number of ARGs at the individual level that were unique to their given infant group and, in addition to the results previously described for visit 1, found significantly fewer unique ARGs in each PS infant compared to the NS infants at visit 2 (*P* = 0.0180) and visit 4 (*P* = 0.0144, *P* = 0.0105) (Figs. 4A, S15A, Additional file 1). When we looked at the matched subset of preterm infants, however, we only found significant differences in the unique number of ARGs at the visit 1 time-point (Figs. S15B, S16A, Additional file 1).

### Antibiotic inactivating genes are more prevalent in preterm infants not supplemented with probiotics

Overall, we identified a reduced diversity of ARGs in both preterm infant groups at visit 4 (5-month corrected age) compared to in early life (Figs. 3, 4B, S15C, Additional file 1). In the NS group of infants, we found more ARGs classified as antibiotic inactivating genes at all time-points compared to their PS counterparts (Figs. 4B, S15C, Additional file 1). This result was corroborated in our matched subset of preterm infants (Figs. S15D, S16B, Additional file 1). Therefore, probiotic supplementation reduced the diversity of antibiotic inactivation genes detected in the preterm infant gut resistome up to 5 months of age.

### Preterm infants not supplemented with probiotics retain certain AMR gene families

We next looked at the diversity of AMR gene families in the preterm infants up to 5 months of age (Figs. S17, S18, Additional file 1). We found certain AMR genes that were unique to the NS infants across many time-points and never identified in the PS infants. These genes belong to the following AMR gene families: *aac(3)*, *bla*_CblA_, *bla*_CTX-M_, *bla*_OXA_, streptogramin vat acetyltransferase, tetracycline inactivation enzymes (*tetX* and *tet(X4)*), and various vancomycin resistance genes (Figs. 4C, S15C, Additional file 1). Also, there were certain AMR gene families that, although they were identified at early time-points in both preterm groups, appeared to persist longer in the NS infants than in the PS infants. These families included the aminoglycoside resistance families *ant(3″)* and *ant(6)*, the SHV-type beta-lactamases, and the fosfomycin thiol transferases (Figs. 4C, S15E). We found similar trends in the matched subset of infants and compared the similarities across both analysis approaches (Figs. S15F, S16C, S17B, S18B, Additional file 1). These results suggests that these preterm infants are exposed to similar ARGs at an early age, but that probiotic supplementation prevents prolonged retention of these ARGs in the gut resistome.

### Genetic context of antibiotic resistance genes was retained in the infant gut

**Fig. 5:**
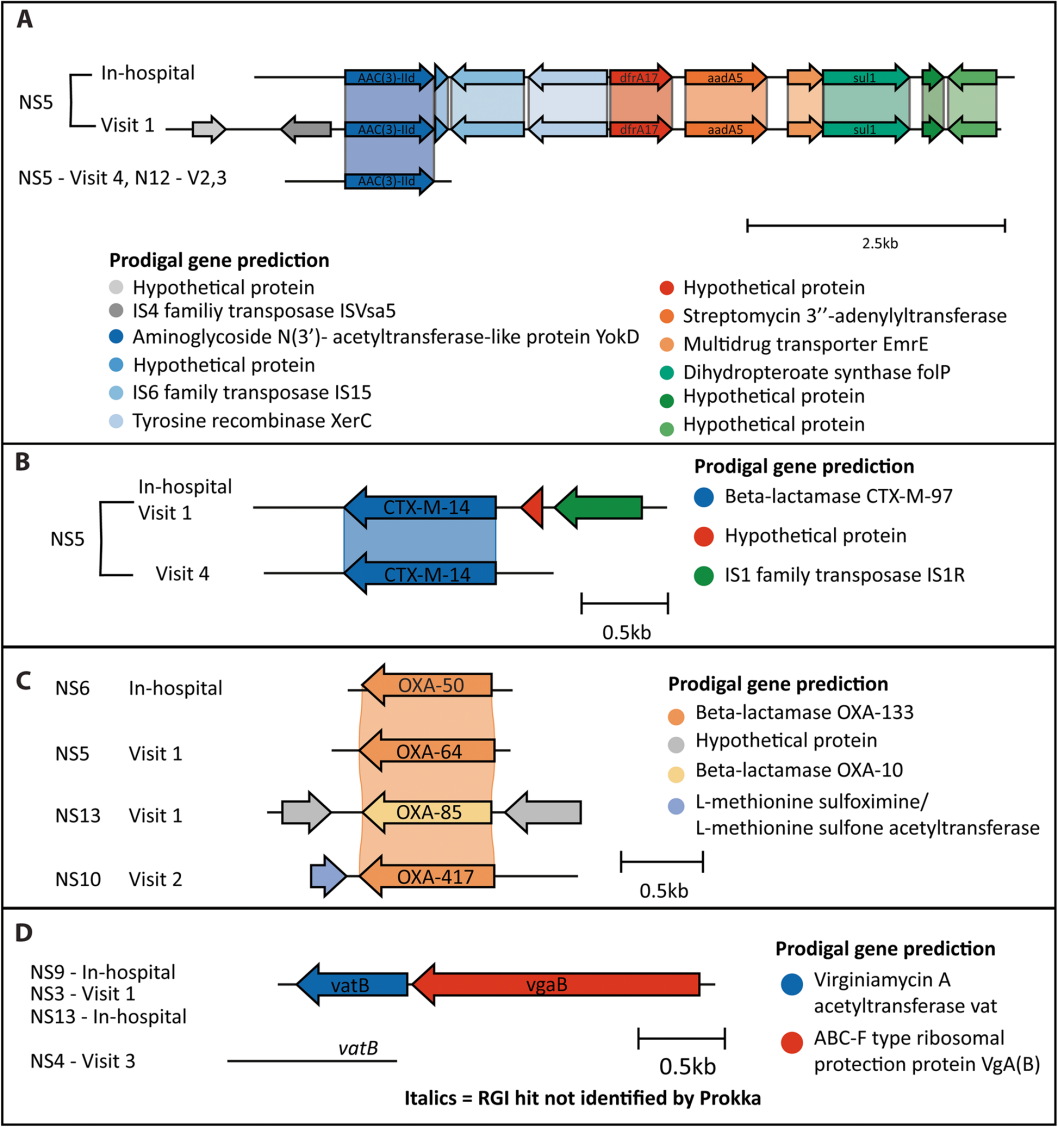
Genetic context of AMR gene families unique to NS infants. From the de novo assembly, open reading frames were annotated using Prokka, and resistance genes were predicted using RGI *main*. The Prokka annotations are the colored arrows, and the RGI *main* predictions are labeled on each ORF. The genes are shown grouped into their respective AMR gene families: (A) AAC(3) gene family, (B) CTX-M beta-lactamase family, (C) OXA beta-lactamase family, (D) streptogramin vat acetyltransferase family (NS = not supplemented preterm)

**Fig. 6:**
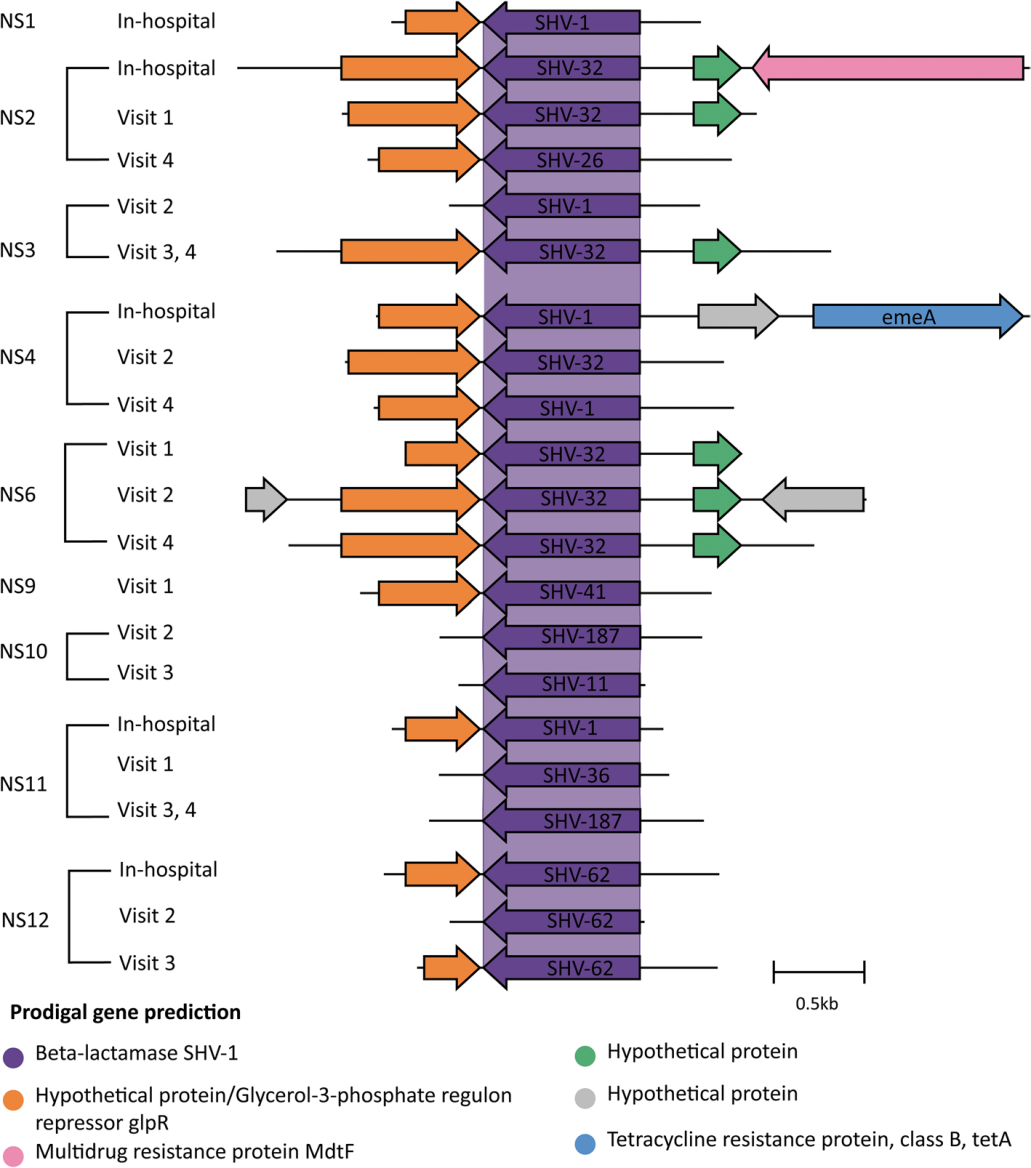
Genetic context of the SHV beta-lactamases. From﻿ the de novo assembly, open reading frames were annotated using Prokka, and resistance genes were predicted using RGI *main*. The Prokka annotations are the colored arrows, and the RGI *main* predictions are labeled on each ORF (NS = not supplemented preterm)

A unique feature of targeted capture is the ability to increase not only the depth of sequencing coverage of ARGs but also the surrounding genes. Using the k-mer-based pathogen-of-origin prediction feature of RGI (RGI *kmer*_query) and MOB-suite’s *mob_recon* algorithm, we identified the genetic context of specific ARGs, predicted potential bacterial hosts of ARGs, and identified MGEs carrying these ARGs in the infant gut microbiome (Figs. 5, 6, S19–S24, Additional files 1 and 4). In many cases for the NS infants, the genetic context of ARGs was conserved within individuals over the various study time-points indicating the persistence of the same host organism or mobile genetic element housing the ARGs. This was also found between individual infants. These cases included *aac(3)-IId* in NS5 and NS12 over multiple time-points, *bla*_CTX-M-14_ in NS5 from in-hospital up to 5 months of age, and various *bla*_SHV_ genes that were detected from in-hospital up to 3 and 5 months of age only in NS infants (Figs. 5AB, 6). With both *aac(3)-IId* and *bla*_CTX-M-14_, the genes were near IS4, IS6, or IS1 family transposases, highlighting the potential mobility of these genes. The contig containing *aac(3)-IId* in infant NS5 at visit 1 is similar to a plasmid identified in *K. pneumoniae* and other *Gammaproteobacteria* based on the MOB-suite analysis (Additional file 4). This contig also contained the ARGs *dfrA17*, *aadA5*, and *sul1*. In other NS and PS infants, these additional genes were found in different genomic contexts and potential plasmids identified in *Gammaproteobacteria*, *Enterobacteriaceae*, and *Enterobacterales*, highlighting the mobility of these ARGs (Fig. S21, Additional files 1 and 4). The SHV beta-lactamases likely originated from *Klebsiella* spp. based on the RGI *kmer_query* results but may be associated with plasmids with a broad host range in *Gammaproteobacteria* and *Enterobacterales* (Additional file 4).

Other clusters of AMR genes were more prevalent in NS infants compared to the PS and FT infants. Vancomycin resistance genes possibly originating from *Enterococcus* spp. were prominent at later time-points in various NS infants and were not identified on plasmids (Fig. S19, Additional files 1 and 4). Tetracycline inactivation enzymes (i.e., *tetX*) flanked by the rRNA methyltransferase *ermD* were detected in NS infants at various time-points, likely originated from *Bacteroides fragilis*, and were not associated with any known plasmids (Fig. S20B, Additional files 1 and 4). CblA beta-lactamases were most similar to sequences from uncultured bacteria or *Bacteroides uniformis* in NCBI, and contigs containing this ARG did not show similarity to plasmids in MOB suite (Fig. S20A, Additional files 1 and 4). Finally, the ANT(6) AMR family, consisting of *ant(6)-Ia*, *aad(6)*, and *aadS*, and the combination of the streptogramin vat acetyltransferase, *vatB* and *vgaB*, were likely found in Gram-positive organisms such as *Staphylococcus aureus* and *Enterococcus* spp. and have been associated with plasmids in these genera (Figs. S22, 5D, Additional files 1 and 4).

For the few genes that were more prominent in the PS infants, the OKP beta-lactamases likely originated from *Klebsiella* spp., based on the RGI *kmer_query* results. Based on the MOB-suite analysis, these genes are likely associated with plasmids with a broad host range in *Gammaproteobacteria*, *Enterobacteriaceae*, and *Enterobacterales* (Fig. S23, Additional files 1 and 4). There are two genes identified from the APH(2″) family: *aph(2*″*)-IIa* and *aph(2*″*)-Iva* (Fig. S24, Additional file 1). From the RGI *kmer_query* analysis, the latter was likely found in *Enterococcus* spp., while the former has a broader host range, including *Clostridiodes* spp. (Additional file 4). Neither were similar to plasmids via the MOB-suite analysis.

Including these highlighted ARGs, over 200 instances of ARGs were predicted to be on a plasmid (Additional file 4). Many of the genes originated from *S. aureus*, including *mecA*, *mecI*, *mecR1*, *ermC*, *msrA*, *mphC*, *bla*_PC1_, *lnuA*, *dfrC*, *qacA*, and *qacB*. These genes were not unique to any infant group nor sample collection time-point. The *mecA*, *mecI*, *mecR1*, and *ermC* genes were more commonly found in NS infants. ARGs potentially found on plasmids from *Enterobacteriaceae* and *Gammaproteobacteria* included *sul2*, *bla*_TEM-1_, *aac(6')-Ib-cr*, *aph(3”)-Ib*, *aph(6)-Id*, *dfrA1*, *E. coli bla*_ampC_, and *tetO*. The *sul2*, *aac(6′)-Ib-cr*, and *aph(6)-Id* genes were more common in NS infants compared to the other infants we investigated.

## Discussion

Given the consequences of antibiotic exposure on the gut microbiome and resistome, and the evidence suggesting that probiotics reduce the risk of NEC in preterm infants, we sought to study the effect of probiotics on the preterm infant gut resistome. While the overall number of ARGs between the groups of infants did not differ significantly, we found differences in the unique types of ARGs and resistance mechanisms. The NS infants harbored more unique ARGs associated with antibiotic inactivation mechanisms of resistance than the PS and FT infants. We also identified ARGs that persisted longer throughout the study period in NS infants compared to PS infants. Finally, by harnessing the unique aspects of targeted capture and our analysis approaches, we could predict potential hosts and plasmid sequences associated with ARGs. Therefore, our survey of the resistome in preterm infants suggests that probiotics given within the first 12 weeks of life reduce the diversity of ARGs in the preterm infant gut and prevent the persistence of ARGs up to 5 months of age.

Antibiotic resistance genes were found in all infant gut microbiome samples, regardless of whether they were preterm or received probiotics. This is not surprising given that a diverse gut resistome containing beta-lactam, tetracycline, aminoglycoside, and chloramphenicol resistance genes has been found in both preterm and full-term infants [21, 69, 70]. We identified many of the same beta-lactamases, aminoglycoside-modifying enzymes, and tetracycline protection proteins as those detected previously in preterm and full-term infants, suggesting similar exposures to ARG-carrying bacteria throughout early life, perhaps from the NICU or other shared environments [8, 11, 12, 22, 41, 43].

We found more unique ARGs in NS preterm infants than in PS preterm infants at visit 1 (term age) and full-term infants at 10 days of age. This contradicts a previous study, where the resistome of preterm infants encoded fewer unique ARGs; however, the relative abundance of these ARGs was higher than in full-term infants [21], highlighting the importance of going beyond enumerating ARGs alone. Few studies have compared probiotic-supplemented preterm infants to full-term infants. One reported a much lower diversity of ARGs in all infants than our study did (99 vs over 200) [43]. This disparity is likely due to the limited detection of antibiotic resistance genes with shallow shotgun metagenomic sequencing. They noted that non-probiotic-supplemented preterm infants had a higher abundance of certain beta-lactamases and efflux pumps compared to preterm infants supplemented with a similar probiotic to our study and full-term infants at 7 days [43]. Another group surveyed the resistome of preterm infants supplemented with *Bifidobacterium longum* subsp. *infantis* EVC001 using much deeper shotgun metagenomics (average 33.6 million reads per sample) and detected 315 unique ARGs [41]. They found that the burden of AMR was lower in PS preterm infants, and 67 unique ARGs, including a chloramphenicol acetyltransferase and macrolide resistance genes, were more abundant in NS infants [41]. One final study, however, reported no significant difference in the resistome of PS and NS preterm infants [42]. This study used the very limited approach of PCR to detect a small set of resistance genes [42].

Our design is distinct from others because we applied a powerful hybridization-based sequencing approach to specifically target over 2000 ARGs with 50–300× less sequencing effort than shotgun metagenomics, which reduces the cost of expanding to larger sample sets. As others have noted, we also found that NS preterm infants had a distinct resistome compared to PS preterm infants and full-term infants. This was evident in the number of unique ARGs we identified and the differences in resistance mechanisms and AMR gene families that were present. While we detected the same genes in our infant samples as other studies [41, 43], we did not highlight these same genes as differences between the infant groups. Alternatively, we found the association of unique antibiotic inactivation genes including the beta-lactamases *bla*_CblA_, *bla*_CTX-M_, and *bla*_OXA_ and aminoglycoside-modifying enzyme *aac(3)* with only the NS infants and a reduced number of unique ARGs in the PS and FT infants. Preterm infants that received probiotics were more comparable to full-term infants than NS infants in terms of the distribution of resistance mechanisms that were detected in the gut microbiome, in particular the numbers of antibiotic efflux and antibiotic inactivation genes. These results suggest that probiotic supplementation with *Bifidobacterium* and *Lactobacillus* species soon after birth reduces the diversity of antibiotic resistance genes in the preterm infant gut, resulting in a resistome that is more similar to full-term infants at 10 days of age than that of other preterm infants.

While previous longitudinal studies have investigated the impact of probiotics on the preterm infant gut resistome up to 4 months of age, we had a later time-point at 5-month corrected age. Esaiassen and co-workers reported that the resistome of PS infants was not significantly different from that of more mature infants at 4 months of age, suggesting that probiotics remediated the effects of premature birth and antibiotic exposure [43]. Nguyen and colleagues did not follow-up with infants after discharge from hospital but found that a longer stay in the NICU resulted in greater accumulation of ARGs, and probiotic supplementation reduced this effect [41]. While we did not have full-term infants to compare to at older time-points, we found that PS infants consistently had fewer unique ARGs up to 5 months of age as compared to NS infants. We, and others, also observed that the diversity of ARGs was higher at earlier points in life and decreased over time in both groups of preterm infants [8, 10]. Others have not noted, as we have, that certain aminoglycoside-modifying enzymes (*aac(3)*) and beta-lactamases (*bla*_CTX-M-14_, *bla*_SHV_) persisted longer in NS infants than PS infants. The reduced diversity and persistence of ARGs associated with probiotic supplementation should be further monitored beyond 5 months of age. An approach similar to ours could facilitate these studies in a cost-effective and sensitive way.

To go beyond what has been accomplished with previous shotgun sequencing studies, we used the unique benefit of targeted capture in increasing the coverage of genetic regions surrounding ARGs to predict potential hosts and MGEs associated with those ARGs. Potentially mobile ARGs were found in all infants, as has been reported previously for both full-term and preterm infants [21, 22, 43, 69]. Indeed, the antibiotic inactivation genes that persisted in the NS infants in our study up to 5-month corrected age (*aac(3)-IId*, *bla*_CTX-M-14_, *bla*_SHV_) were associated with MGEs in various MDR *Enterobacteriaceae* and enterococci (Additional file 4) [71–73]. Bacteria belonging to the Enterobacteriaceae family have proportionately high levels of ARGs and can facilitate the transfer of ARGs through MGEs to other pathogens [73–77]. The association of these genes with MGEs and resistant pathobionts highlights the potential risk of dissemination of ARGs in the preterm infant gut. Various ARGs associated with MGEs and *Enterobacteriaceae* were more common in NS infants than PS infants and therefore suggest probiotics reduce the diversity of potentially mobile antibiotic resistance.

Similar to another study, we detected vancomycin ARGs in infants that did not receive vancomycin at later collection time-points as well as the *mecA* gene (Fig. S19, Additional files 1 and 4) [43]. We captured the entire vancomycin resistance gene cluster (consisting of 5 genes) in NS infants at visit 3 (12-week corrected age) and 4 (5-month corrected age) that likely originated from *Enterococcus gallinarum* or other enterococci (Fig. S19, Additional files 1 and 4). Methicillin-resistant *Staphylococus aureus* (MRSA) harbor the staphylococcal cassette chromosome *mec* that consists of the genes *mecA*, *mecI*, and *mecR1* [78]. The combination of two or more of these genes on the same contig was identified in 3 NS infants and 1 full-term infant in our study (Additional file 4). *Enterococcaceae* and *Staphylococcaceae* are often reported in preterm infants; however, only a few infants in our study had a relative abundance of > 1% of these families (Fig. S4, Additional file 1) [3, 6, 9, 10, 21, 79, 80]. Despite the low abundance of these families of bacteria, we still detected ARGs that likely originated from *Staphylococcus* spp. and *Enterococcus* spp*.* at various time-points. These two examples highlight the sensitivity of our approach and the potential to monitor rates of vancomycin-resistant enterococci or MRSA colonization and infection. We did not try isolating these organisms to test their antibiotic susceptibility.

Another interesting result is the prevalence and persistence of the SHV beta-lactamases in NS infants (Fig. 6). SHV beta-lactamases confer intrinsic resistance to penicillins and first-generation cephalosporins and are core chromosomal genes in a group of *Klebsiella pneumoniae* [73, 81, 82]. These genes have since been mobilized on plasmids in other members of the *Enterobacteriaceae*. The presence of SHV beta-lactamases at multiple study time-points in NS infants could suggest competitive inhibition of certain *K. pneumoniae* strains by the probiotic bacteria. We detected other genes that likely originated from *Klebsiella* spp. including *bla*_OKP_ in PS infants and therefore cannot rule out the absence of *K. pneumoniae* in this infant group. Indeed, in the microbiota of the subset of infants, various ASVs associated with strains of *K. pneumoniae* were detected, although they were more abundant in NS infants. This may be a result of exposures to different *K. pneumoniae* strains in the NICU by these two groups of preterm infants.

Strengths of our study were its longitudinal nature, where samples were collected both in-hospital and after discharge (up to 5-month corrected age) and the timing of our study that captured a change in protocol in the NICU to provide probiotic supplementation as standard procedure. The sensitivity of our sequence capture method allowed us to detect ARGs at low prevalence with a small amount of sequencing data, and the ability to enrich the genetic context of ARGs allowed us to predict potential hosts and mobilization of ARGs. The limitations of our study included the small number of samples at each time-point and variability in antibiotic exposures of the infants. Compared to other approaches, targeted capture does not reflect the microbiome’s functional genes or species diversity and cannot detect previously uncharacterized antibiotic resistance genes [46, 83, 84]. Finally, both analysis approaches used in our study have limitations when detecting antibiotic resistance in metagenomes [85] and rely on a reference database that requires curation and frequent updates. In general, more ARGs belonging to the AMR mechanism group of antibiotic efflux and antibiotic inactivation were reported in all infants. This reflects the biased distribution of resistance genes curated in the CARD, which is itself based on ARGs reported in the scientific literature [46].

## Conclusions

In this study of preterm infants, we have highlighted the potential of a *Bifidobacterium* spp. and *Lactobacillus* spp. containing probiotic to reduce the diversity of AMR in the preterm infant gut. When compared with probiotic-supplemented infants, infants that did not receive probiotics had a higher number of unique ARGs that were predominantly associated with the mechanism of antibiotic inactivation and a greater diversity of antibiotic resistance genes that persisted up to 5 months of age in the gut microbiome. Furthermore, we highlight how the unique combination of targeted capture and analysis approaches can resolve individual ARGs, their surrounding genetic context, and predict potential bacterial hosts. This allowed us to associate many of the persistent antibiotic resistance genes in non-probiotic-supplemented infants with *Enterobacteriaceae* and MGEs. Our results suggest that probiotics can be used as a supplement during hospitalization to reduce the diversity of AMR in preterm infants that are exposed to a variety of multidrug resistant pathogens at an early age. Our study highlights the feasibility and advantages of using targeted capture in longitudinal cohort studies of the resistome and why it should be considered to further improve preterm infant care.

## Supplementary Information

**Additional file 1: Figure S1.** Antibiotic exposure of preterm infants. **Table S1.** Information regarding the full-term infants. **Table S2.** Information regarding the subset of preterm infants. **Figure S2.** Sample collection and probiotic exposure of the subset of infants. **Figure S3.** Antibiotic exposure of the subset of preterm infants. **Figure S4.** Relative abundance of bacterial families in the infant gut microbiome. **Figure S5.** Targeted capture of resistance genes in the full set of infants. **Figure S6.** Targeted capture of resistance genes in the subset of infants. **Figure S7.** Differences in the resistome identified through RGI main at Visit 1. **Figure S8.** Distribution of genes detected at the AMR gene family level through RGI bwt. **Figure S9.** Distribution of genes detected at the AMR gene family level through RGI main. **Figure S10. **Differences in the resistome identified through RGI bwt in the subset at Visit 1. **Figure S11.** Differences in the resistome identified through RGI main in the subset at Visit 1. **Figure S12.** Unique genes in each infant group at various timepoints for the preterm infants. **Figure S13.** Unique genes in each infant group for the subset of preterm infants at various timepoints. **Figure S14.** Unique genes in each infant group at various timepoints for the subset of preterm infants. **Figure S15.** Unique ARGs, mechanisms, and families identified in preterm infants through RGI main. **Figure S16.** Unique ARGs, mechanisms, and families identified in the subset of preterm infants through RGI bwt. **Figure S17.** AMR gene families identified through RGI bwt. **Figure S18.** AMR gene families identified through RGI main. **Figure S19.** Genetic context of vancomycin resistance gene families detected in all infants. **Figure S20.** Genetic context of AMR families more prominent in NS infants. **Figure S21.** Genetic context of ANT(3”) resistance gene families detected in all infants. **Figure S22.** Genetic context of the ANT(6) gene family in all infants. **Figure S23.** Genetic context of OKP beta-lactamases detected in all infants. **Figure S24.** Genetic context of the APH(2”) gene family detected in all infants.

**Additional file 2.** Supplementary Methods.

**Additional file 3.** Results of enrichment for ARGs – Details on library preparation, enrichment, sequencing and analysis results for each sample.

**Additional file 4.** Predicted bacterial host of ARGs – For a subset of ARGs, the bacterial host was predicted through various analysis approaches.

**Additional file 5.** Negative control results – Results of negative controls included throughout the workflow and sequencing results for one sample.

## Data Availability

Sequence data that support the findings of this study have been deposited in NCBI’s Sequence Read Archive with the BioProject accession code: PRJNA805248. Two participants (NS2 and NS5) did not consent to the release of their data. Code used to analyze the data is available at https://github.com/AllisonGuitor/AMR-metatools or https://zenodo.org/badge/latestdoi/444553774.

## Abbreviations

NICU: Neonatal intensive care unit
MDR: Multidrug resistant
ARG(s): Antibiotic resistance gene(s)
NEC: Necrotizing enterocolitis
PCR: Polymerase chain reaction
Baby and Pre-Mi: Baby and Preterm Microbiota of the Intestine Cohort Study
PS: Probiotic supplemented
NS: Not supplemented
FT: Full term
RGI: Resistance Gene Identifier
CARD: Comprehensive Antibiotic Resistance Database
qPCR: Quantitative PCR
ASV: Amplicon sequence variant
AMR: Antimicrobial resistance
RGI *bwt*: Metagenomic read-mapping feature of RGI
RGI *kmer_query*: Pathogen-of-origin feature of RGI
RGI *main*: Genome annotation feature of RGI
ORF(s): Open reading frame(s)
MGE(s): Mobile genetic element(s)
PMA: Postmenstrual age
ESBLs: Extended-spectrum beta-lactamases
