# Supplementary material for: Capturing the antibiotic resistome of preterm infants reveals new benefits of probiotic supplementation
Source: Microbiome. 2022 Aug 26;10:136. doi: 10.1186/s40168-022-01327-7 (PMC9414150; doi:10.1186/s40168-022-01327-7)
Supplement: Supplementary file 3 — Additional file 2. Supplementary Methods. [file 40168_2022_1327_MOESM2_ESM.docx]

**Supplementary Methods**

**Capturing the antibiotic resistome of preterm infants reveals new benefits of probiotic supplementation**

**Allison K. Guitor**^1-3^, Efrah I. Yousuf^4^, Amogelang R. Raphenya^1-3^, Eileen K. Hutton^5,6^, Katherine M. Morrison^4,6^, Andrew G. McArthur^1-3^, Gerard D. Wright^1-3^, Jennifer C. Stearns^1,5-8^.

^1^Department of Biochemistry and Biomedical Sciences, McMaster University

^2^Michael G. DeGroote Institute for Infectious Disease Research, McMaster University

^3^David Braley Centre for Antibiotic Discovery, McMaster University

^4^Department of Pediatrics, McMaster University

^5^Department of Obstetrics & Gynecology, McMaster University

^6^The Baby & Mi and the Baby & Pre-Mi Cohort studies

^7^Department of Medicine, McMaster University

^8^ Farncombe Family Digestive Health Research Institute, McMaster University

**Library preparation methods -** The numbers correspond to values used in File S1.

1. No size-selection, only a clean-up step during library preparation;
   10 cycles of PCR amplification;
   Final library eluted in 33 ul 0.1XTE
2. No size selection, only a clean-up step during library preparation;
   8 cycles of PCR amplification;
   Final library eluted in 33 ul 0.1XTE
3. Size selection for 500-700 bp inserts
   7 cycles of PCR amplification;
   Final library eluted in 33 ul 0.1XTE.
4. No size selection, only a clean-up step during library preparation;
   7 cycles of PCR amplification;
   Final library eluted in 33 ul 0.1XTE
5. Size selection for 500-700 bp inserts
   4 cycles of PCR amplification;
   Final library eluted in 33 ul 0.1XTE.
