# Supplementary material for: Capturing the antibiotic resistome of preterm infants reveals new benefits of probiotic supplementation
Source: Microbiome. 2022 Aug 26;10:136. doi: 10.1186/s40168-022-01327-7 (PMC9414150; doi:10.1186/s40168-022-01327-7)
Supplement: Supplementary file 2 — Additional file 1: Figure S1. Antibiotic exposure of preterm infants. Table S1. Information regarding the full-term infants. Table S2. Information regarding the subset of preterm infants. Figure S2. Sample collection and probiotic exposure of the subset of infants. Figure S3. Antibiotic exposure of the subset of preterm infants. Figure S4. Relative abundance of bacterial families in the infant gut microbiome. Figure S5. Targeted capture of resistance genes in the full set of infants. Figure S6. Targeted capture of resistance genes in the subset of infants. Figure S7. Differences in the resistome identified through RGI main at Visit 1. Figure S8. Distribution of genes detected at the AMR gene family level through RGI bwt. Figure S9. Distribution of genes detected at the AMR gene family level through RGI main. Figure S10. Differences in the resistome identified through RGI bwt in the subset at Visit 1. Figure S11. Differences in the resistome identified through RGI main in the subset at Visit 1. Figure S12. Unique genes in each infant group at various timepoints for the preterm infants. Figure S13. Unique genes in each infant group for the subset of preterm infants at various timepoints. Figure S14. Unique genes in each infant group at various timepoints for the subset of preterm infants. Figure S15. Unique ARGs, mechanisms, and families identified in preterm infants through RGI main. Figure S16. Unique ARGs, mechanisms, and families identified in the subset of preterm infants through RGI bwt. Figure S17. AMR gene families identified through RGI bwt. Figure S18. AMR gene families identified through RGI main. Figure S19. Genetic context of vancomycin resistance gene families detected in all infants. Figure S20. Genetic context of AMR families more prominent in NS infants. Figure S21. Genetic context of ANT(3”) resistance gene families detected in all infants. Figure S22. Genetic context of the ANT(6) gene family in all infants. Figure S23. Genetic context of OKP beta-l [file 40168_2022_1327_MOESM1_ESM.docx]

Figure S1: Antibiotic exposure of preterm infants.

The antibiotics preterm infants were exposed to throughout the duration of the study. A colored box indicates that infant received the antibiotic. The dots within each box represent the number of times they were given that antibiotic. The length of treatment is not reflected in this figure.

Table S1: Information regarding the full-term infants.

|  | **Full-term (n = 9)** |
| --- | --- |
| Gestational age at birth, weeks | 39.78 ± 0.61 |
| Probiotic exposure, weeks | None |
| Antibiotic exposure during sample collection (types and number of infants exposed) | None |
| Antibiotic exposure, weeks | None |
| In-hospital sample, weeks in PMA (N) | N/A |
| Visit 1 sample, weeks in PMA (N) | 41.25 ± 0.64 (9) |
| Visit 2 sample, weeks in PMA (N) | N/A |
| Visit 3 sample, weeks in PMA (N) | N/A |
| Visit 4 sample, weeks in PMA (N) | N/A |
| PMA is the postmenstrual age in weeks and SD is the standard deviation. The data are presented as mean ± SD. | |

Table S2: Information regarding the subset of preterm infants.

|  | **NS preterm**  **(n = 6)** | **PS preterm**  **(n = 6)** | ***p*-value** |
| --- | --- | --- | --- |
| Gestational age at birth, weeks | 28.26 ± 0.84 | 27.81 ± 1.65 | 0.60 |
| Probiotic exposure, weeks | 0 | 8.21 ± 3.68 | **<0.0001** |
| Antibiotic exposure during sample collection (types and number of infants exposed) | Amo(1) Amp(6) Cefa(2) Cefo(2) Cl(1) G(6) V(1) | Amp(6) Cefa(1) Cefo(2) Cl(3) G(6) Met(2) V(1) | N/A |
| Antibiotic exposure, weeks | 1.02 ± 0.80 | 1.48 ± 1.17 | 0.49 |
| In-hospital sample, weeks in PMA (N) | 36.69 ± 3.84 (6) | 37.86 ± 1.69 (3) | 0.96 |
| Visit 1 sample, weeks in PMA (N) | 41.03 ± 1.86 (5) | 42.38 ± 1.88 (6) | 0.31 |
| Visit 2 sample, weeks in PMA (N) | 47.26 ± 2.23 (5) | 46.43 ± 0.50 (5) | >0.99 |
| Visit 3 sample, weeks in PMA (N) | 50.19 ± 1.33 (3) | 54.21 ± 1.85 (6) | **0.04** |
| Visit 4 sample, weeks in PMA (N) | 61.81 ± 1.95 (6) | 59.68 ± 0.62 (4) | 0.08 |
| PMA is the postmenstrual age in weeks and SD is the standard deviation. The data are presented as mean ± SD. | | | |

Figure S2: Sample collection and probiotic exposure of the subset of infants.

Timelines from birth to final sampling point for the subset of infants. The duration of exposure to probiotics (lavender bar) as well as all study points included in this study (up to 4 timepoints) for not supplemented (NS) and probiotic-supplemented (PS) preterm infants as well as full-term (FT) infants are shown in relation to postmenstrual age in weeks.

Figure S3: Antibiotic exposure of the subset of preterm infants.

The antibiotics preterm infants were exposed to throughout the duration of the study. A colored box indicates that infant received the antibiotic. The dots within each box represent the number of times they were given that antibiotic. The length of treatment is not reflected in this figure.

Figure S4: Relative abundance of bacterial families in the infant gut microbiome.

Stacked bar charts representing the relative abundance of bacterial families in the subset of preterm infants up to 5 months corrected age and for full-term infants at 10 days postpartum. NS = not supplemented preterm, PS = probiotic-supplemented preterm, and FT = full-term infants. The category <1% abundance represents all ASVs that were present at less than 1% of the relative abundance across all infants.

Figure S5: Targeted capture of resistance genes in the full set of infants.

A) A subsampled number of reads were mapped to CARD using RGI *bwt*. The percentage of reads mapping to the target of ARGs is reported for each infant at Visit 1. Significant differences are indicated by a line and asterisk(s) above the groups compared. P = 0.0014 for no probiotic vs full-term. B) Using the results of mapping reads to CARD, the number of ARGs with at least 100 mapped reads is reported for Visit 1. C) Subsampled reads from each infant sample were subjected to *de novo* assembly and prediction of ARGs using RGI *main*. The number of genes predicted at each Visit 1 is reported. D) The percentage of reads mapping to the target of ARGs is reported for preterm infants across all visits. E) Using the results of mapping reads to CARD, the number of ARGs with at least 100 mapped reads is reported for preterm infants at all visits. F) Subsampled reads from each infant sample were subjected to *de novo* assembly and prediction of ARGs using RGI *main*. The number of genes predicted in each preterm infant at all visit timepoints is reported.

Figure S6: Targeted capture of resistance genes in the subset of infants.

A) For the subset of preterm infant samples and full-term infants at all visits, a subsampled number of reads were mapped to CARD using RGI *bwt*. The percentage of reads mapping to the target of ARGs is reported for each infant at Visit 1. Significant differences are indicated by a line and asterisk(s) above the groups compared. P = 0.0318 for Visit 1 no probiotic vs full-term. B) Using the results of mapping reads to CARD, the number of ARGs with at least 100 reads is reported for all infants at Visit 1. C) Subsampled reads from each infant sample were also subjected to *de novo* assembly and prediction of ARGs with RGI *main*. The number of genes predicted at Visit 1 is reported. D) The percentage of reads mapping to the target of ARGs is reported for preterm infants at all visits. E) Using the results of mapping reads to CARD, the number of ARGs with at least 100 reads is reported for all preterm infants at all visits. F) Subsampled reads from each infant sample were also subjected to *de novo* assembly and prediction of ARGs with RGI *main*. The number of genes predicted at all visits for the preterm infants is reported.

**Figure S7:** **Differences in the resistome identified through RGI *main* at Visit 1.**

These ARG counts are from the results of *de novo* assembly and ARG prediction using RGI *main* and CARD. Perfect and strict hits are reported. The data presented is from all preterm and full-term infants at Visit 1. A) Unique and overlapping ARGs identified in each infant group. The number of infant samples included in each is shown next to the sample type. B) The number of unique ARGs identified in each infant. Significant differences are denoted by a line and asterisk(s) above the groups that were compared. P = 0.0052 for NS vs PS, P = 0.0012 for PS vs FT. C) A breakdown of the mechanisms of antibiotic resistance identified in each infant group. The number of infant samples included in each is shown next to the sample type. D) The presence or absence of selected AMR gene families in each infant group. A teal box indicates that at least one gene from that AMR gene family was identified in any of the infant samples. NS = not supplemented preterm, PS = probiotic-supplemented preterm, and FT = full-term infants.

Figure S8: Distribution of genes detected at the AMR gene family level through RGI *bwt*.

A teal box indicates that at least one gene from that AMR gene family was identified in any of the infant samples at that timepoint through read mapping to CARD. A) For all preterm and full-term infants. B) For the subset of preterm infants and all full-term infants.

Figure S9: Distribution of genes detected at the AMR gene family level through RGI *main*.

A teal box indicates that at least one gene from that AMR gene family was identified in any of the infant samples at that timepoint through *de novo* assembly and prediction of ARGs through RGI *main*. A) For all preterm and full-term infants. B) For the subset of preterm infants and all full-term infants.

**Figure S10:** **Differences in the resistome identified through RGI *bwt* in the subset at Visit 1.**

Reads were mapped to CARD using bowtie2 and antibiotic resistance genes with at least 100 reads were reported. The data presented is from the subset of preterm infants and all full-term infants at Visit 1. A) Unique and overlapping ARGs identified in each infant group. The number of infant samples included in each is shown next to the sample type. B) The number of unique ARGs identified in each infant. Significant differences are denoted by a line and asterisk(s) above the groups that were compared. P = 0.0040 for NS vs PS, P = 0.0203 for PS vs FT. C) A breakdown of the mechanisms of antibiotic resistance identified in each infant group. The number of infant samples included in each is shown next to the sample type. D) The presence or absence of selected AMR gene families in each infant group. A teal box indicates that at least one gene from that AMR gene family was identified in any of the infant samples. NS = not supplemented preterm, PS = probiotic-supplemented preterm, and FT = full-term infants.

**Figure S11:** **Differences in the resistome identified through RGI *main* in the subset at Visit 1.**

These ARG counts are from the results of *de novo* assembly and ARG prediction using RGI *main* and CARD. Perfect and strict hits are reported. The data presented is from the subset of preterm infants and all full-term infants at Visit 1. A) Unique and overlapping ARGs identified in each infant group. The number of infant samples included in each is shown next to the sample type. B) The number of unique ARGs identified in each infant. Significant differences are denoted by a line and asterisk(s) above the groups that were compared. P = 0.0404 for NS vs FT, P = 0.0102 for NS vs PS, P = 0.0024 for PS vs FT. C) A breakdown of the mechanisms of antibiotic resistance identified in each infant group. The number of infant samples included in each is shown next to the sample type. D) The presence or absence of selected AMR gene families in each infant group. A teal box indicates that at least one gene from that AMR gene family was identified in any of the infant samples. NS = not supplemented preterm, PS = probiotic-supplemented preterm, and FT = full-term infants.

Figure S12: Unique genes in each infant group at various timepoints for the preterm infants.

These gene counts are from the results of *de novo* assembly and ARG prediction using RGI *main* and CARD. Perfect and strict hits are reported. Data from all preterm infants the in-hospital collection (A), Visit 1 (B), Visit 2 (C), Visit 3 (D), and Visit 4 (E) timepoints. The number of infants included in each time point is indicated. NS = not supplemented preterm, PS = probiotic-supplemented preterm.

Figure S13: Unique genes in each infant group for the subset of preterm infants at various timepoints.

These gene counts are from the results of mapping reads to CARD using RGI *bwt* and counting the number of genes with at least 100 reads. Data from the subset of preterm infants at the in-hospital collection (A), Visit 1 (B), Visit 2 (C), Visit 3 (D), and Visit 4 (E) timepoints. The number of infants included in each time point is indicated. NS = not supplemented preterm, PS = probiotic-supplemented preterm.

Figure S14: Unique genes in each infant group at various timepoints for the subset of preterm infants.

These gene counts are from the results of *de novo* assembly and ARG prediction using RGI *main* and CARD. Perfect and strict hits are reported. A) Data from the subset of preterm infants and all full-term infants at all time points. B) Data from the subset of preterm infants and all full-term infants at Visit 1. C-H) Data from the subset of preterm infants at all time points, in-hospital collection, Visit 1, Visit 2, Visit 3, and Visit 4. The number of infants included in each time point is indicated. NS = not supplemented preterm, PS = probiotic-supplemented preterm, and FT = full-term infants.

Figure S15: Unique ARGs, mechanisms, and families identified in preterm infants through RGI *main*.

These ARG counts are from the results of *de novo* assembly and ARG prediction using RGI *main* and CARD. Perfect and strict hits are reported. Figures A, C, and E represent the full set of preterm infants. Figures B, D, and F represent the subset of preterm infants. AB) The number of unique ARGs identified in each infant. Significant differences are denoted by a line and asterisk(s) above the groups that were compared. P = 0.0040 for Visit 1, P = 0.0180 for Visit 2, P = 0.0105 for Visit 4 for panel A. P = 0.0089 for Visit 1 in panel B. CD) The number of ARGs identified in each infant group classified by resistance gene mechanism. The number of infant samples included in each is shown next to the sample type. NS = non-probiotic-supplemented preterm, PS = probiotic-supplemented preterm. EF) A selected subset of detected AMR gene families in preterm infants. A teal box indicates that at least one gene from that AMR gene family was identified in any of the infants at that time-point.

Figure S16: Unique ARGs, mechanisms, and families identified in the subset of preterm infants through RGI *bwt*.

Reads were mapped to CARD using RGI *bwt* and antibiotic resistance genes with at least 100 reads were reported. The data presented is from the subset of preterm infants at all visits. A) The number of unique ARGs identified in each infant. Significant differences are denoted by a line and asterisk(s) above the groups that were compared. P = 0.0102 for Visit 1. B) The number of ARGs identified in each infant group classified by resistance gene mechanism. The number of infant samples included in each is shown next to the sample type. NS = non-probiotic-supplemented preterm, PS = probiotic-supplemented preterm. C) A selected subset of detected AMR gene families in preterm infants. A teal box indicates that at least one gene from that AMR gene family was identified in any of the infants at that time-point.

Figure S17: AMR gene families identified through RGI *bwt*.

A teal box indicates that at least one gene from that AMR gene family was identified in any of the infant samples at that timepoint through read mapping to CARD. Each column is an individual study timepoint and the figure is divided between infants that received probiotics and those that did not. A) Results for the full set of preterm infants. B) Results for the subset of preterm infants.

Figure S18: AMR gene families identified through RGI *main*.

A teal box indicates that at least one gene from that AMR gene family was identified in any of the infant samples at that timepoint through *de novo* assembly and prediction with RGI *main*. Each column is an individual study timepoint and the figure is divided between infants that received probiotics and those that did not. A) Results for the full set of preterm infants. B) Results for the subset of preterm infants.

Figure S19: Genetic context of vancomycin resistance gene families detected in all infants.

From the *de novo* assembly, open reading frames were annotated using Prokka and resistance genes were predicted using RGI *main*. The Prokka annotations are the colored arrows and the RGI *main* predictions are labelled on each ORF. NS = not supplemented preterm and PS = probiotic-supplemented preterm.

Figure S20: Genetic context of AMR families more prominent in NS infants.

From the *de novo* assembly, open reading frames were annotated using Prokka and resistance genes were predicted using RGI *main*. The Prokka annotations are the colored arrows and the RGI *main* predictions are labelled on each ORF. A) CblA beta-lactamase family. B) tetracycline inactivation enzymes. NS = not supplemented preterm, PS = probiotic-supplemented preterm, and FT = full-term infants.

Figure S21: Genetic context of ANT(3’’) resistance gene families detected in all infants.

From the *de novo* assembly, open reading frames were annotated using Prokka and resistance genes were predicted using RGI *main*. The Prokka annotations are the colored arrows and the RGI *main* predictions are labelled on each ORF. NS = not supplemented preterm, PS = probiotic-supplemented preterm, and FT = full-term infants.

Figure S22: Genetic context of the ANT(6) gene family in all infants.

From the *de novo* assembly, open reading frames were annotated using Prokka and resistance genes were predicted using RGI *main*. The Prokka annotations are the colored arrows and the RGI *main* predictions are labelled on each ORF. NS = not supplemented preterm and PS = probiotic-supplemented preterm.

Figure S23: Genetic context of OKP beta-lactamases detected in all infants.

From the *de novo* assembly, open reading frames were annotated using Prokka and resistance genes were predicted using RGI *main*. The Prokka annotations are the colored arrows and the RGI *main* predictions are labelled on each ORF. NS = not supplemented preterm, PS = probiotic-supplemented preterm, and FT = full-term infants.

Figure S24: Genetic context of the APH(2’’) gene family detected in all infants.

From the *de novo* assembly, open reading frames were annotated using Prokka and resistance genes were predicted using RGI *main*. The Prokka annotations are the colored arrows and the RGI *main* predictions are labelled on each ORF. NS = not supplemented preterm and PS = probiotic-supplemented preterm.
